# Vitamin K Dependent Proteins in Kidney Disease

**DOI:** 10.3390/ijms20071571

**Published:** 2019-03-29

**Authors:** Ciprian N. Silaghi, Tamás Ilyés, Vladimir P. Filip, Marius Farcaș, Adriana J. van Ballegooijen, Alexandra M. Crăciun

**Affiliations:** 1 Department of Molecular Sciences, University of Medicine and Pharmacy “Iuliu Hațieganu”, 400012 Cluj-Napoca, Romania; tamasilyes94@gmail.com (T.I.); vladimirpfilip@gmail.com (V.P.F.); farcasmarius47@gmail.com (M.F.); acraciun@umfcluj.ro (A.M.C.); 2 Department of Nephrology & Epidemiology and Biostatistics, Amsterdam University Medical Center, VUmc, 1117 HV Amsterdam, The Netherlands; aj.vanballegooijen@vumc.nl

**Keywords:** Vitamin K dependent proteins, matrix Gla protein, osteocalcin, Gas6, Gla-rich protein, chronic kidney disease, calcification

## Abstract

Patients with chronic kidney disease (CKD) have an increased risk of developing vascular calcifications, as well as bone dynamics impairment, leading to a poor quality of life and increased mortality. Certain vitamin K dependent proteins (VKDPs) act mainly as calcification inhibitors, but their involvement in the onset and progression of CKD are not completely elucidated. This review is an update of the current state of knowledge about the relationship between CKD and four extrahepatic VKDPs: matrix Gla protein, osteocalcin, growth-arrest specific protein 6 and Gla-rich protein. Based on published literature in the last ten years, the purpose of this review is to address fundamental aspects about the link between CKD and circulating VKDPs levels as well as to raise new topics about how the interplay between molecular weight and charge could influence the modifications of circulating VKDPs at the glomerular level, or whether distinct renal etiologies have effect on VKDPs. This review is the output of a systematic literature search and may open future research avenues in this niche domain.

## 1. Introduction

Chronic kidney disease (CKD) has a major influence on the quality of life in many patients. Therefore, it is important to develop early intervention methods for better management of complications and to decrease CKD mortality. While there are various markers used to assess the stages of CKD, the effectiveness of treatment is low since none of these newly discovered molecules and mechanisms improve disease progression [1,2].

Vitamin K comprises a group of fat-soluble vitamins that act as co-factor for γ-glutamyl carboxylase, which activates several vitamin K-dependent proteins (VKDPs). VKDPs play a major role in hemostasis and bone health, but also in the progression of vascular calcification (VC), which is strongly associated with cardiovascular risk [3] and CKD mortality [4,5]. Several VKDPs also have important roles as ligands in apoptotic pathways with a significant implication in cancer therapy and disease progression [6,7,8]. The four most acknowledged extrahepatic VKDPs we selected for this review are: matrix Gla protein (MGP), osteocalcin (OC), growth arrest specific protein 6 (Gas6) and Gla-rich protein (GRP).

The activity of VKDPs is influenced by their carboxylation status, thus being dependent on vitamin K level. Vitamin K deficiency, assessed by the uncarboxylated conformations of MGP and OC was found to be common in patients with CKD [9] and hemodialysis (HD) [10]. To date, there is no gold standard to measure vitamin K sufficiency, therefore an assessment of different circulating VKDPs and monitoring dietary intake are applied.

To our knowledge, no reviews were published in the past ten years on the significance of MGP, OC, Gas6 and GRP in CKD. Taking into consideration the growing state of knowledge and the fast pace of research in this field, we aim to offer a comprehensive analysis of studies from the past ten years (cross-sectional, prospective cohort, meta-analyses and interventional studies) with attention being directed towards understanding the molecular mechanisms of VKDPs in CKD. We analyzed the implication of the four VKDPs in CKD, hemodialysis (HD), peritoneal dialysis, renal transplant, acute renal injury, nephrolithiasis, renal cancer or related in-vitro studies. We also approached new topics on the interaction between molecular weight and charge of VKDPs at glomerular level, about the influence of CKD etiologies upon VKDPs and the relationship between circulating VKDPs levels and the progression of CKD.

## 2. Methodology

### 2.1. Search Strategy and Selection of Studies

All databases accessible through the PubMed search engine were selected for this review. Human, as well as animal and in vitro studies were taken into consideration, but only articles published in the past ten years were selected for screening. The set of search terms was jointly agreed upon by the authors, and subsequently used during the initial selection process. The search terms are summarized in Table 1.

Each search term was introduced into the search engine, then a list was made for each set of results and the date when the databases were accessed was noted. The lists for each protein were cross-checked for duplicates between search terms and then duplicates were subsequently removed. From these lists, only articles written in English and only those that had abstracts were reviewed. After reviewing the abstracts, full text articles were obtained for each eligible article, which were assessed according to the inclusion and exclusion criteria.

### 2.2. Inclusion and Exclusion Criteria

The inclusion and exclusion criteria were jointly agreed upon by the authors. The inclusion criteria were as follows: written in the past 10 years, written and published in English, concerning CKD, hemodialysis, peritoneal dialysis, renal transplant, renal cancer, acute renal injury, nephrolithiasis or related in-vitro cellular studies. Exclusion criteria were: reviews, systematic reviews, genetic analysis studies, studies concerning the effects of vitamin K antagonists, studies concerning hormonal effects and/or treatment, studies concerning vitamin D supplementation, studies concerning immunosuppressive treatment, inability to determine the exact type of protein studied and studies that were lacking clear method definition.

Most studies were related to CKD, defined as the reduction of kidney filtration, estimated by the glomerular filtration rate (eGFR). Decreasing eGFR denoted a directly proportional decrease in kidney filtration capacity, being the main criteria by which CKD was classified into stages. Stage V of CKD was also referred to as end-stage renal disease (ESRD) [11].

### 2.3. Identification, Selection, Screening and Inclusion

**Figure 1:**
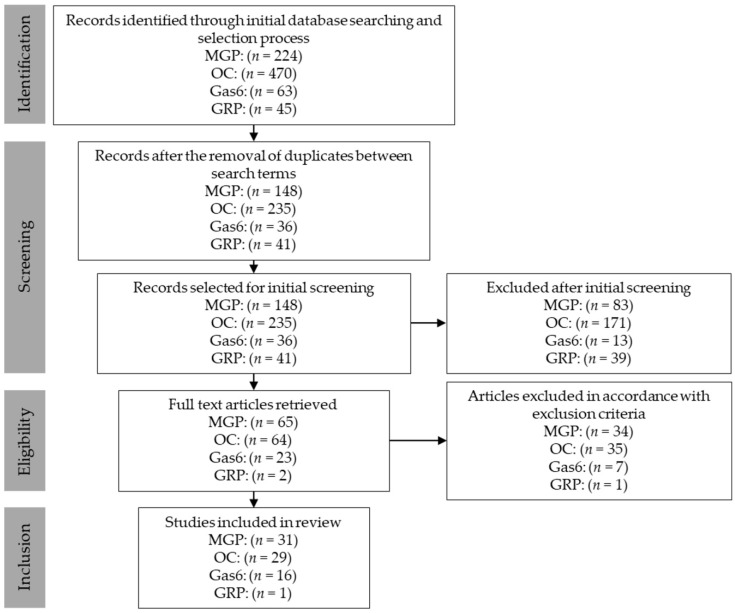
Flow diagram of the processes from identification, selection and screening to inclusion. Abbreviations: MGP, matrix Gla protein; OC, osteocalcin; Gas6, growth arrest specific protein 6; GRP, Gla-rich protein.

The process of identification, selection, screening and inclusion of the studies are depicted in Figure 1. The following studies were included: MGP [12,13,14,15,16,17,18,19,20,21,22,23,24,25,26,27,28,29,30,31,32,33,34,35,36,37,38,39,40,41,42]; OC [9,10,43,44,45,46,47,48,49,50,51,52,53,54,55,56,57,58,59,60,61,62,63,64,65,66,67,68,69]; Gas6 [70,71,72,73,74,75,76,77,78,79,80,81,82,83,84,85]; and GRP [86].

## 3. Functional and Molecular Background

### 3.1. Matrix Gla Protein

MGP is a 10.6 kDa protein, comprised of 84 amino acids which contains nine glutamic acid (Glu) residues but also five serine (Ser) residues, being insoluble in water [87]. MGP is synthesized and secreted in the extracellular matrix mainly by vascular smooth muscle cells (VSMCs) and chondrocytes [88].

To exert its biological role as calcification inhibitor, MGP needs to be fully carboxylated and in phosphorylated conformation (p-cMGP). With the contribution of vitamin K as cofactor [89], the carboxylation of the five Glu residues in positions 2, 37, 41, 48 and 52 is facilitated by γ-glutamyl carboxylase, becoming γ-carboxy glutamic acid (Gla) residues. In addition, three Ser residues are phosphorylated in positions 3, 6 and 9 with the help of the casein kinase [90,91]. These post-translational extrahepatic modifications ensure the normal function of MGP as VC inhibitor.

There is strong evidence suggesting that carboxylated MGP (cMGP) inhibits VC by binding Ca^2+^ ions via the –COOH groups from the Gla residues [87]. cMGP also binds to hydroxyapatite molecules, while uncarboxylated MGP (ucMGP) was associated with progression of VC [92,93]. Moreover, dephospho-uncarboxylated MGP (dp-ucMGP), the inactive conformation, was associated with increased VC in patients with ESRD [12]. There are also available assays that cannot discriminate between different conformations of MGP, designated as total MGP (t-MGP); however, these assays have not yet been studied in CKD [94].

In rats, the calcium clearance was accomplished by a high molecular weight circulating complex consisting of MGP, fetuin-A, hydroxyapatite and other proteins [95] The same study postulated that serum proteins of 10-kDa (such as MGP) are cleared rapidly by kidney filtration (serum half-life = 5 min), the increase of MGP serum level being caused by new synthesis of MGP and that γ-carboxylation is necessary for its binding to this circulating complex. As regards to MGP, this mineral complex has not yet been confirmed in humans, MGP exerting its activity as VC inhibitor in tissues, not in circulation.

**Figure 2:**
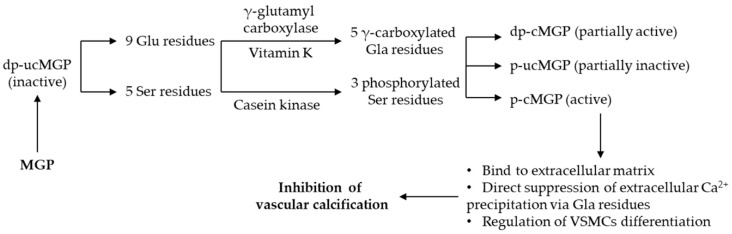
Molecular mechanisms and physiological function of MGP. Abbreviations: MGP, matrix gla protein; Glu, glutamic acid; Gla, γ-carboxy-glutamic acid; Ser, serine; dp-ucMGP, dephospho-uncarboxylated MGP; p-ucMGP, phosphorylated-uncarboxylated MGP; p-uMGP, phosphorylated-carboxylated MGP; VSMCs, vascular smooth muscle cells.

The molecular mechanisms of MGP and the main physiological function as inhibitor of VC are summarized in Figure 2 [12,87,89,90,91,92,93,96].

### 3.2. Osteocalcin

While MGP is produced mainly by VSMCs, OC is secreted exclusively in bone by osteoblasts, only a small part of it diffuses into the systemic circulation [97]. Having a molecular weight of 5.6 kDa and containing around 49 amino acids [97], this water soluble protein is the smallest molecule among the four VKDPs. OC undergoes similar post translational vitamin K dependent γ-carboxylation as MGP [98]. Depending on carboxylation status, OC presents two possible conformations: uncarboxylated OC (ucOC) and carboxylated OC (cOC) with three Gla residues. Different variables (e.g., age and menopausal status) may influence the circulating OC fractions. In this respect, both circulating ucOC and cOC were higher in older women, but only ucOC was correlated with advancing age [99].

Besides bone dynamics, where OC is a validated marker of bone turnover, it is also a hormone that regulates glucose metabolism [100,101]. It was demonstrated that ucOC, the active endocrine conformation, stimulates insulin secretion either directly in the pancreas or indirectly by means of increasing glucagon-like peptide-1 secretion in the small intestine, as well as release of adiponectin in adipose tissue, therefore enhancing insulin sensitivity in muscle [100]. Moreover, insulin signaling in osteoblasts was found to be a positive modulator for OC expression but also for its activation through the capability to enhance bone resorption indirectly by osteoclasts [101].

When bone is resorbed, OC fragments are released into the circulation, thus plasma concentrations may reflect bone turnover. The clearance of OC in humans is through glomerular filtration (plasma half-life = 20 min), the levels of serum intact OC being higher in patients with CKD compared with age-matched controls [102].

For the relationship between OC and VC, a consistent association was reported [98]. Circulating ucOC is proposed as marker of subclinical atherosclerosis in non-dialysis patients with CKD, because lower circulating levels of ucOC could discriminate between patients and a healthy population [103].VSMCs from calcified atheroma plaques have also been found to significantly express OC, especially in HD patients [98,104].

**Figure 3:**
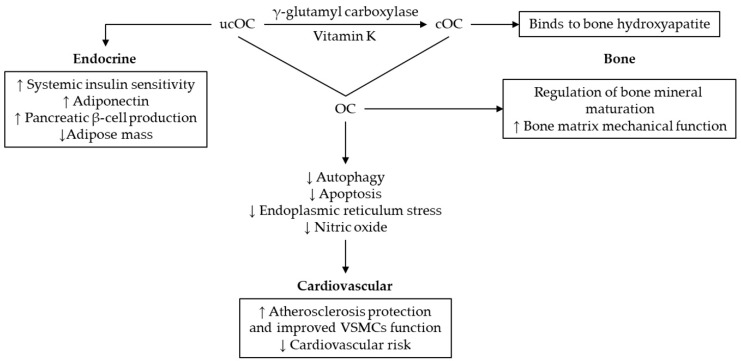
Systemic and bone specific effects of OC. Abbreviations: OC, osteocalcin; ucOC, uncarboxylated OC; cOC, carboxylated OC; VSMCs, vascular smooth muscle cells.

The systemic and bone specific effects of OC are summarized in Figure 3 [97,100,105,106,107,108].

### 3.3. Growth Arrest Specific Protein 6

Gas6, a protein with a N-terminal Gla domain, has a molecular weight of 75 kDa, but an idiosyncratic function among VKDPs, being involved in multiple physiological activities ranging from cellular homeostasis, cell proliferation to cell survival [109,110,111]. Similar to other VKDPs, the carboxylation is carried out with the help of vitamin K dependent γ-glutamyl carboxylase. Because of its carboxylated Gla domain, the Ca^2+^ binding activity increases, leading to multiple protein-protein interactions. Cells that express the Gas6 gene vary from leukocytes and endothelial cells to VSMCs, with the assumption that Gas6 would act as a growth factor-like molecule in the proliferation of VSMCs [112].

Structurally and functionally related to Protein S, Gas6 has been shown to be a ligand for the TAM receptor family (Tyro3-Axl-Mer) [6,113]. These tyrosine-kinase receptors are key controllers of cell survival and proliferation processes. Dysregulation in any of its molecular components leads to evasion of cancer immune system and drug resistance mechanisms [8]. Studies that investigated the Gas6/TAM pathway in cancer therapy highlighted its importance, but the exact mechanism is still elusive [114]. Gas6 and soluble Axl are present in human circulation at nanomolar level concentrations [115].

**Figure 4:**
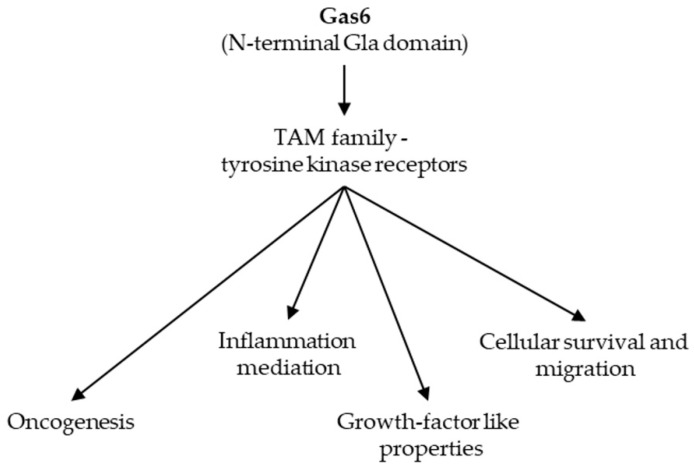
General mechanism of action for Gas6. Abbreviations: Gas6, growth arrest specific protein 6; TAM, Tyro3-Axl-Mer.

The general mechanism of action for Gas6 is summarized in Figure 4 [6,8,113,114].

### 3.4. Gla-Rich Protein

The etymology of GRP, the most recently discovered member of the VKDPs family, originates from its large number of Gla residues [116]. GRP has a molecular weight of 10.2 kDa and contains 74 amino acids of which sixteen are Gla residues, yet being insoluble at neutral pH [117]. GRP is involved in the inhibition of articular and cardiovascular calcifications [118,119]. It is mainly found in skin, bone vasculature and cartilage, where it functions as a calcification inhibitor [119,120].

Since GRP was associated with the presence of calcification, it might prevent calcium-induced signaling pathways, leading to inhibition of calcium crystal formation [117,121,122]. The mechanisms for calcium binding are similar to MGP; its high number of –COOH containing Gla residues enhance GRP’s ability to bind large amounts of calcium ions, even more than other VKDPs. Moreover, GRP has been shown to have the same capability of binding to hydroxyapatite crystals as MGP [122].

## 4. VKDPs in Kidney Disease

### 4.1. Vitamin K Insufficiency in Kidney Disease

Vitamin K insufficiency has been found to be present in the majority of CKD and HD patients [9,10], however the origin for the poor vitamin K status in these populations has not yet been fully elucidated. The only conceivable reason found was an apparent lower vitamin K intake related to the dietary routine recommended in CKD and HD, which limits sodium and potassium and consequently reduces the intake of foods with high vitamin K content [10].

The higher levels of plasma dp-ucMGP found in patients with CKD and HD validates the theory of a poor vascular vitamin K status, which is an underlying cause for VC and cardiovascular related mortality in these patients [10,13,14,15,42]. Furthermore, considering the assessment of the total OC percentage that is not carboxylated (%ucOC), a subclinical vitamin K deficiency was found in 60% of patients with CKD stages III–V [9].

**Figure 5:**
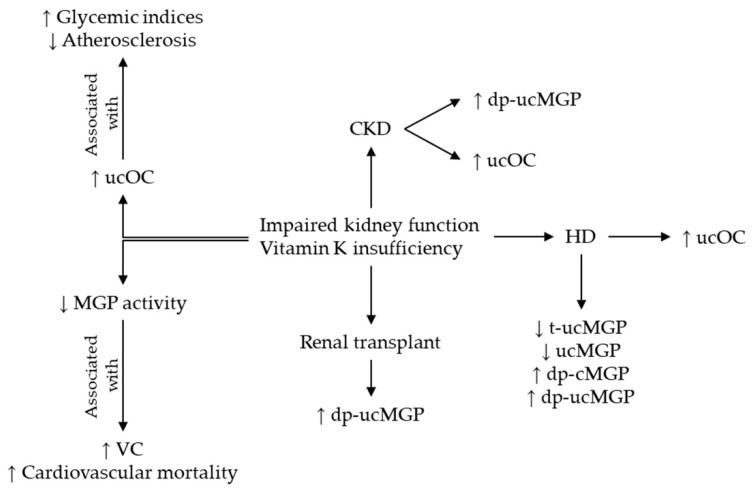
Modification of circulating MGP and OC in CKD. Abbreviations: CKD, chronic kidney disease; OC, osteocalcin, ucOC, uncarboxylated OC; cOC = carboxylated OC; MGP, matrix Gla protein; dp-ucMGP, dephospho-uncarboxylated MGP; t-ucMGP, total-uncarboxylated MGP; ucMGP, uncarboxylated MGP; dp-cMGP, dephospho-carboxylated MGP.

Figure 5 summarizes the modifications of different MGP and OC conformations in CKD and the effects of vitamin K insufficiency upon these proteins [9,10,12,13,14,15,16,17,18,19,20,21,22,23,24,25,26,42,43,44].

### 4.2. Matrix Gla Protein in Kidney Disease

#### 4.2.1. Human Studies on Circulating MGP

Studies analyzing the variation of circulating MGP levels in patients with kidney disease are summarized in Table 2. A glance at the studies using dual-antibody assays (dp-ucMGP or dp-cMGP) shows that all of the circulating MGP conformations were higher in patients with different CKD stages, HD or renal transplant compared to a healthy population. In these studies, the levels of dp-ucMGP are higher with CKD stages and were associated with different localizations of VC. However, no association was found between plasma dp-ucMGP levels and calcified carotid intima media thickness in patients with diabetic nephropathy (DN) [27]. On the contrary, the serum MGP conformations using mono-antibody assays, such as ucMGP and t-ucMGP (a modified assay after ucMGP), were found decreased in the same pathologies in adults [25] and children [26]. Cranenburg et al. [25] showed that serum ucMGP concentrations were inversely associated with the extent of VC in HD patients, but in non-diabetic HD patients ucMGP was not associated with VC [28].

How can MGP modifications be explained in the context of these conflicting results? None of the studies mentioned above have assessed total MGP levels, thus low serum ucMGP may reflect either a reduced MGP production or a poor carboxylation status for ucMGP due to vitamin K insufficiency in CKD and HD patients. It is noteworthy that assays using ucMGP or t-ucMGP cannot detect the phosphorylated conformations of MGP or its fragments. A hypothesis was proposed by Cranenburg et al. [25] that ucMGP binds to the areas of calcification by its negatively phospho-Ser residues. To support this hypothesis, the levels of circulating phosphorylated MGP were lower in patients with highly calcified CKD [17]. Therefore, due to the lack of phospho-Ser residues, dp-ucMGP could be delivered more rapidly than ucMGP into the bloodstream.

In addition, different responses after vitamin K supplementation were reported for the MGP species in patients with CKD: plasma levels of dp-ucMGP decreased after vitamin K_2_ supplementation [15,38], while vitamin K_1_ intake was not associated with circulating ucMGP levels [39]. Kurnatowska et al. [38] noticed that administration of vitamin K_2_ in patients with CKD stages III–V did not have an effect on the progression of VC. On the contrary, treatment with lanthanum carbonate (a non-calcium and non-aluminum based phosphate binder) in maintenance HD patients with diabetes and adynamic bone disease could delay the progress of coronary artery calcification, showing a significant decrease in serum MGP compared to baseline [40].

#### 4.2.2. MGP in Experimental Studies

There are also in vitro studies assaying MGP levels in different supernatants from calcifying VSMCs incubated in serum of HD patients. The results are summarized in Table 3.

Willy et al. [30] found that serum samples from patients after High Cut-Off dialysis (HCO) have a reduced capacity to induce in vitro calcification in VSMCs compared to patients after conventional High Flow dialysis (HF). A possible explanation of this decline in pro-calcifying activity could be that, after incubation with serum from HF, VSMCs produced considerably higher anti-calcifying proteins (e.g., MGP) than VSMCs incubated in HCO or Medium Cut-Off dialysis (MCO) serums, as a compensatory response to the augmented calcification environment. In addition, according to Willy et al. [29], High Retention Onset dialyzers (HRO) may offer a treatment option to diminish the progression of VC, but they come with significant albumin loss. As an alternative, the HRO with a steeper cut-off in their dialysis membrane are faintly less permeable, sustaining the desired effect on in vitro calcification, meanwhile limiting the albumin loss.

To sustain the anti-calcifying function of MGP, it was demonstrated that oxalate and hydroxyapatite crystals are able to provoke renal epithelial cells to produce MGP in an induced nephrolithiasis model on both young rats and tissue culture [31,32]. Khan et al. [31] stated that contact of renal epithelial cells to oxalate generates reactive oxygen species and consequently oxidative stress. Thus, the enhanced expression of MGP by exposure to reactive oxygen species leads to atherogenic stimuli. Several studies were conducted in adults and children [123] to find a better marker for oxidative stress and it would be an interesting direction for future studies to assess the relationship between MGP and oxidative stress in patients with kidney disease.

#### 4.2.3. Studies Assessing MGP in Tissues

In Table 4, the studies on the assessment of MGP in tissues are presented.

In all the studies on tissues in which the presence of calcifications was validated by von Kossa staining, ucMGP and cMGP were present nearby the calcification sites.

Shroff et al. [36] found increased amounts of ucMGP compared with cMGP in dialysis vessels, whereas control vessels had predominantly cMGP. The presence of MPG conformations was evocative for the vesicle-mediated calcification process. The study also demonstrated that hydroxyapatite crystals were released from dead VSMCs, thus confirming their role to initiate calcification.

Moreover, tissue from patients with CKD (e.g., interstitial nephritis) revealed calcium deposits with both ucMGP and cMGP conformations constantly co-localized with the micro-calcifications [37]. On the contrary, in normal renal tissue, the micro-calcifications were absent and the staining for both MGP conformations was negative. The study per se was first to demonstrate local vitamin K deficiency in renal tissue collected from CKD patients compared to healthy donors.

### 4.3. Osteocalcin in Kidney Disease

#### 4.3.1. Osteocalcin in CKD and Renal Transplant

Studies addressing the variations of serum OC levels in human subjects with CKD and renal transplant are summarized in Table 5.

As a common complication in CKD, the secondary hyperparathyroidism was assessed in a large cohort by parathyroid hormone and OC as marker of bone turnover. The higher levels of OC reported in post-renal transplant patients with CKD stage IV versus those with CKD III [46] are in agreement with the findings of Holden et al. [9], even when they assessed %ucOC. In addition, post-renal transplant patients with higher levels of serum OC showed an increase bone turnover [50]. Contrary, lower OC levels were found in CKD stages IV–V than healthy controls, with a non-significant decreasing trend as CKD advanced [45]. The apparent conflicting results obtained by Gluba-Brzózka et al. [45] came from the fact that no statistical significance has been reached when OC was compared to the healthy population.

In terms of the relationship between OC and kidney damage markers, Holden et al. [9] found a strong positive association among %ucOC, stage of CKD and urinary loss of proteins. Circulating OC was inversely correlated with eGFR in patients with CKD stage II–IV [49], as well as with creatinine clearance in older adults without CKD [47]. Although these correlations have been found, they should be analyzed through the perspective of the coexistence of secondary hyperparathyroidism, impairment of glomerular filtration and age-related co-morbidities (bone diseases, VC, cancer, and chronic inflammation). All these determinants could influence the circulating OC levels, complicating the interpretation of the OC results in CKD patients with comorbidities.

There are also interesting findings from cross-sectional studies that open new research directions especially because OC is thought to have hormonal functions. In this respect, higher leptin levels were associated with lower levels of OC in a large cohort of post renal transplant patients [48]. In patients with CKD stage II–IV, higher adiponectin levels were associated with increased serum OC levels, showing a possible OC regulation on pancreatic β-cell proliferation and adipocyte gene expression, causing an increase of insulin secretion and consequently adiponectin synthesis [49]. Therefore, adipose tissue seems to exert an effect on bone tissue, perhaps mediated via leptin and adiponectin secretion. Kovesdy et al. [48] assumed that leptin, a hormone secreted by adipose tissue, probably influences the bone turnover directly by modulating osteoblast differentiation, but also indirectly through the activation of hypothalamic β-adrenergic system and up-regulation of β-adrenergic receptors in bone cells.

#### 4.3.2. Osteocalcin in Dialysis and Interventional Studies

Lower serum OC levels were strongly associated with higher incidence of cardiovascular events in HD patients [43], being also considered a possible diagnostic tool for adynamic bone disease in predialysis patients with ESRD [44]. In a cross-sectional study on HD patients with higher bone turnover, serum OC was higher, while in HD patients with mineralization defects were lower [53], suggesting a possible use of circulating OC as marker of bone formation in HD patients. Moreover, fetuin-A, a systemic calcification inhibitor and modulator of bone metabolism, was negatively associated with serum OC levels in HD patients with high bone turnover [54]. In addition, in line with its hormonal effects, increased levels of serum ucOC were inversely correlated with indices of glucose metabolism in HD patients [55].

Interestingly, serum OC was not associated with bone mineral density in patients with peritoneal dialysis [56]. However, lower OC levels were associated with increased incidence of VC, while serum OC levels were inversely correlated with aortic pulse wave velocity [57,58].

With respect to carboxylation status of OC, surprisingly, both the ucOC and cOC levels were higher in HD patients [10]. There are two possible theories for this finding: either circulating OC fragments are retained in uremic serum of HD patients or the associated ESRD enhanced bone turnover, leading to an overall increase in OC synthesis and consequently to high levels of both cOC and ucOC.

Studies on OC related to treatments and drug administration are summarized in Table 6.

### 4.4. Gas6 in Kidney Disease

The TAM receptors and Gas6 as ligand are essential modulators of complex metabolic processes, ranging from vascular atherosclerosis, thrombosis to inflammation, but few studies are published on Gas6-Axl pathway in humans with CKD [70].

#### 4.4.1. Gas6 in CKD and Acute Kidney Disease

In patients with HD and CKD, Gas6 levels were found to be elevated and inversely associated with eGFR [70]. The most likely reason for the increase of Gas6 is related to its role in endothelial cell function. In specific conditions, such as inflammation and repair, Gas6 and its receptors are expressed by leukocytes and endothelial cells. A study in mice showed that Gas6 is essential in promoting and accelerating the sequestration of leukocytes and platelets in damaged endothelium [124]. Weiner et al. [125] emphasized that endothelial cells in CKD are exposed to specific stress leading to accelerated vascular disease and high cardiovascular mortality. Therefore, a constellation of factors acting on endothelial cells, such as structural degeneration, endothelial damage and oxidative stress, may provoke vascular disruption and inflammation in glomerular capillaries but also in large vessels, influencing the evolution of CKD and peripheral vascular disease, consequently leading to increased Gas6 levels.

Elevated plasma Gas6 levels were also found in patients with Hantaan virus that causes hemorrhagic fever, being directly correlated with the disease severity [71]. This increase is probably attributed to the incurring acute renal injury as the disease takes its course, higher levels being released from endothelial cells into circulation as renal lesions advance.

Patients with DN and micro-/macroalbuminuria were found to have significantly lower Gas6 levels compared to DN patients with normoalbuminuria, and higher Gas6 levels were associated with lower risk for DN [72]. The authors did not provide any hypothesis for those important findings, but they clearly emphasized that Gas6 could be involved in the development and progression of DN, being a potential marker for its early diagnosis.

#### 4.4.2. Gas6 in Renal Cancer

An additional field in which Gas6 shows potential interest is renal cancer, more specifically in clear cell renal carcinoma (CCRC), the most common type of renal cell carcinoma. Inactivation of the Gas6/Axl pathway in CCRC was shown to reverse metastatic and invasive cell phenotypes [73], while Gas6 mediated activation of CCRC cells decreased their viability and migratory capacity [74]. CCRC patients with lower levels of tumor Axl receptor expression and higher levels of tumor Gas6 showed significantly increased survival rate [75]. It was also found that Gas6 activation of Axl was enhanced in the presence of Sunitinib (a tyrosine kinase receptor inhibitor), which might contribute to CCRC cell chemoresistance [76]. Based on the above-mentioned CCRC studies, we can draw the following conclusions: Gas6-mediated activation of Axl in CCRC cells leads to receptor down-regulation and decreased migratory capacity cell-viability, but the Gas6/Axl pathway has no effect on invasion.

#### 4.4.3. Gas6 in Experimental Studies

The majority of studies concerning Gas6 are still at the level of fundamental research. However, the implication of Gas6 in VC and apoptosis has been supported lately in few research papers. In this respect, VSMCs treated with iron citrate showed increased VC due to down-regulation of Gas6/Axl pathway with subsequent prevention of apoptosis in rats [77], while mice treated with aldosterone showed increased Gas6 expression [78]. Administration of Raloxifene (an estrogen receptor modulator) was found to significantly decrease Gas6 levels in aortic valves of rats, with down-regulation of Gas6/Axl pathway and decreased aortic valve calcification [79].

Recombinant mouse Gas6 reduced vascular calcification in rats and showed improvement of acute kidney injury with increased survival rate in septic mice [80,81]. Recombinant mouse Gas6 administration in mice with renal ischemia also showed reduced apoptosis and inflammation of renal tissue [82]. The results from these studies strongly suggest a beneficial role of Gas6 in kidney disease, at least in the mammal models. Gas6 was found to be detectable in the urine of mice with podocyte proliferation in response to acute kidney injury [83]. Renal tubular Gas6 expression was also found to increase in mice under treatment with Captopril [84], Gas6 levels being higher in the early stages of salt-dependent hypertension in rats [85].

Even if inconsistent results have been reported on the implication of Gas6 in kidney disease, we can assume that the pathways of Gas6 and Axl are complex and most likely context-specific, thus their role in VC, apoptosis and inflammation of renal tissue can be challenging.

### 4.5. GRP in Kidney Disease

Although there are several studies on GRP with respect to other pathologies, the field of kidney research is still at an early stage. Only one study met our inclusion criteria for GRP, the newest member of the VKDPs family [86].

Serum calciprotein particles are recently discovered colloidal protein-mineral particles, mainly composed of calcium, phosphate, fetuin-A and calcium-binding proteins, which are believed to possess a defense mechanism against calcium-phosphate precipitation in blood [126]. These particles were found to have lower concentration of GRP in patients with CKD stage V, while in vitro, the formation of calcium-phosphate crystals was greatly reduced by incubation with γ-carboxylated GRP [86]. Even if the particles were associated to CKD calcification predisposition, the precise information about their structure, packaging process and function are missing. Knowing that calciprotein particles are key players for the high calcification potential of CKD uremic serum, Viegas et al. [86] demonstrated, both in vivo and in vitro, that GRP, as a constitutive component of calciprotein particles, is an important inhibitory factor to halt systemic and local calcification. They also indicated the pathogenic pathway of these particles in uremic serum, proving that calciprotein particles with lower levels of GRP and fetuin-A can induce calcification of VSMCs by promoting osteochondrogenic differentiation and inflammation.

The discovery of serum calciprotein particles opens new research horizons for other VKDPs that might enter in their assembly, thus the ratio between the percentages of each VKDP could demonstrate similarities with the interplay found in lipoprotein particles.

## 5. Discussions

Taking into consideration the outcomes of the studies encompassed in this review, it is necessary to raise few topics that could be of interest for future research.

### 5.1. The Interplay between Molecular Charge and Weight Could Play a Role in Glomerular Filtration of VKDPs

Serum concentrations of VKDPs are influenced by multiple determinants ranging from vitamin K status, presence of underlying metabolic and cardiovascular disease to impaired kidney function. The exact mechanism of how CKD contributes to variations of circulating VKDPs levels is not yet entirely understood. Considering the existence of an impairment in glomerular filtration, and tubular injuries being less frequently invoked in patients with CKD [127], we hypothesize that variations in circulating levels of VKDPS are most likely influenced by glomerular filtration. Two essential aspects of VKDPs structure may account in this interplay: molecular weight and molecular charge.

The glomerular membrane is equipped with pores that physiologically do not allow the filtration of serum proteins. Moreover, due to the presence of numerous heparan sulfate proteoglycan molecules in its structure, the glomerular membrane displays a net negative charge [128] that creates an effect of electrostatic repelling with similarly charged proteins. VKDPs possess a variety of Glu and Ser residues which in turn become carboxylated and/or phosphorylated, respectively. These post-translational modifications may allow VKDPs to exhibit a greater negative charge, thus a more enhanced repelling effect at the glomerular membrane level.

Both MGP and GRP have similar molecular weights of 10.6 kDa and 10.2 kDa, respectively [87,117]. On the other hand, OC is the smallest among the four VKDPs with a size of 5.6 kDa [97], while Gas6 is the largest, weighing 75 kDa [109], with a similar molecular weight to albumin (around 66 kDa) [129], which can pass into urine leading to albuminuria in cases of glomerular dysfunction [72]. There are no comparative studies on glomerular filtration with respect to the size of the four molecules of VKDPs. However, two studies tested filtration through dialysis membranes, but only MGP was assessed among the four VKDPs [29,30]. Both studies observed that, after incubation with conventional HF serum, VSMCs produced significantly higher concentrations of MGP than VSMCs under HCO, MCO and HRO serums. It can be concluded that the type of dialyzer influenced the protein expression pattern of VSMCs.

While the molecular weights for MGP and GRP are similar, their net molecular charges vary greatly due to the different number of Gla residues. In contrast to the highly carboxylated GRP, which accounts for a total of sixteen Gla residues, MGP has five Gla residues in its fully carboxylated conformation [87,117]. Gas6 has only one N-terminal Gla domain with eleven potential Gla residues, while OC contains three Gla residues [97,109].

Considering the previously mentioned molecular characteristics, the serum levels of VKDPs should vary considerably in context of CKD progression because glomerular filtration becomes less restrictive as CKD progresses. Smaller molecules, e.g., OC, should be filtered more rapidly, compared to larger ones, such as Gas6. Molecular charge should also play a distinctive role in influencing each protein’s capacity to cross the glomerular barrier. Another aspect worth considering is that the status of carboxylation and phosphorylation for each VKDP contributes to its net negative charge, which results in greater electrostatic repelling for the fully carboxylated and phosphorylated species at the glomerular level.

Instead, VKDPs act differently in terms of circulating modifications. Serum dp-ucMGP levels were found to be elevated in CKD patients [17,18], as well as in HD patients [12,19,20], but ucMGP levels were decreased in HD patients [25,26]. In addition, serum GRP levels were decreased in advanced stages of CKD [86]. Serum OC levels were found elevated in more advanced CKD of post renal transplant patients [46], as well as both cOC and ucOC in HD patients [10]. Gas6 levels were inversely correlated with eGFR, serum levels of Gas6 increasing as CKD stage became more advanced [70].

As mentioned above, vitamin K insufficiency was found to be present in CKD and HD patients [9,10] due to an apparent lower vitamin K intake [10]. Vitamin K insufficiency could be admitted to be present in both CKD and HD patients to approximately the same extent considering that vitamin K is lipophilic and cannot be cleared by dialysis. Accordingly, the circulating levels of uncarboxylated species should increase in these patients, thus VKDPs would be less negatively charged, leading to lower glomerular repelling and consequently to lower circulating levels. Only the decrease of circulating ucMGP [25,26] and GRP (although its uncarboxylated conformation was not assessed) [86] supports this hypothesis, whereas increased dp-ucMGP and ucOC are against [10,17,18]. There is also an alternative explanation, as follows: due to the lack of phospho-Ser residues and therefore less attractiveness to calcification areas, dp-ucMGP would be released more rapidly than ucMGP into circulation, leading to higher serum dp-ucMGP levels.

If we were to introduce solubility in the equation, the overall picture would become more complicated. As mentioned in the Section “Functional and Molecular Background”, MGP and GRP are insoluble in water, only OC being water-soluble. According to our knowledge, there are no human studies published so far on the assessment of VKDPs in urine. However, a strong positive correlation between plasma Gas6 levels and urinary Gla residues in patients with liver diseases has been found [130]. In addition, Gas6 was detected in the urine of mice with podocyte proliferation in response to acute kidney injury [83].

In context of normal renal function, OC and its fragments are rapidly cleared by the kidney, contributing to the pool of urinary Gla residues. Rathore et al. [131] supported the idea that OC fragments accumulate and are detectable in renal failure, proposing urinary OC as future marker of bone turnover regardless of its limitations as age related renal impairment or higher pre-analytical variability compared to OC serum assays.

Overall, to test the hypothesis of glomerular repelling and its interplay with the size of VKDPs, future studies should analyze and compare the circulating concentration to the urinary output for each VKDP.

### 5.2. The Relationship between the Etiologies of CKD and the Modifications of Circulating VKDPs

Because the pathology of CKD encompasses multiple etiologies such as DN, nephrolithiasis, autoimmune nephritis and others, we must raise the question whether the variation in circulating VKDPs levels are caused by the impaired renal function itself or by the underlying etiologies of CKD.

Most of the studies either did not identify the specific etiology of the kidney disease [9,14,17,18,45,46,80,86] or in the study protocol the authors included multiple etiologies that were all considered as CKD [15,38] Moreover, the statistics used for the assessment of association between VKDPs and etiologies of CKD are missing. Most of the studies found associations between VKDPs and CKD progression or parameters of kidney damage [9,15,41], but did not perform any correlations with the etiology itself or at least with subgroup of etiologies. This may suggest that circulating VKDP levels are in fact influenced by the accompanying renal impairment, rather than by distinct etiologies responsible for CKD.

Over the past ten years, we found only two studies that actually analyzed VKDPs in a specific etiology related to CKD [27,72]. In the first study, lower Gas6 levels were found in patients with DN and micro-/macroalbuminuria compared to DN patients with normoalbuminuria, higher Gas6 levels being associated with lower risk for DN [72]. However, Roumeliotis et al. [27] found no association between plasma dp-ucMGP levels and calcified carotid intima media thickness in patients with DN.

Due to missing data, a clear conclusion cannot be confidently drawn. However, since the modifications of circulating VKDPs could also be interpreted as an epiphenomenon of the etiology itself, future longitudinal studies are warranted to validate the etiology as a determinant among others that satellite CKD, such as: vitamin K insufficiency, secondary hyperparathyroidism or impairment of glomerular filtration.

### 5.3. VKDPs as Potential Markers in Kidney Disease

The outcomes of studies encompassed in this review can show us the utility of VKDPs as screening tools in kidney disease. It has been reported that circulating levels of dp-ucMGP [17,18], Gas6 [70], OC and %ucOC [9,46] increased with the advance of CKD stages. In this respect, we can rule out GRP, because there is only one study performed in patients with CKD without referring to disease progression [86].

Apart from the fact that circulating dp-ucMGP was demonstrated to increase with CKD progression, reaching a peak in patients with HD, dp-ucMGP levels were also positively associated with the severity of VC in patients with CKD [17]. In line with this evidence, serum levels of ucMGP were inversely associated with the extent of VD in HD patients [25]. On the other hand, phosphorylated MGP conformations had generally low circulating levels in patients with highly calcified CKD, thus limiting their use as a biomarker [17].

It is not clear if serum MGP levels, mainly attributed to the inactive dp-ucMGP, are influenced directly by kidney disfunction or more so by vitamin K insufficiency. The cross-sectional and cohort studies only assessed dp-ucMGP levels in CKD, but not serum levels of total MGP [94]. The increase in dp-ucMGP levels might only be a shift in the ratio of active versus inactive forms of MGP, with total MGP levels remaining unchanged. However, there are also longitudinal studies which validated the relationship between dp-ucMGP and HD, concluding that lower levels of circulating dp-cMGP could be designated as possible predictor of mortality in HD patients [12].

Moreover, different responses after vitamin K supplementation were demonstrated for dp-ucMGP and ucMGP. The circulating dp-ucMGP levels declined after vitamin K_2_ supplementation [15,38] in CKD patients, while vitamin K_1_ supplementation was not associated with circulating ucMGP levels in HD patients [39]. Therefore, ucMGP level was a weak surrogate for functional vitamin K_1_ deficiency, while circulating dp-uc MGP responded in a correlated manner with the supplementation of vitamin K_2_, thus reflecting vitamin K_2_ status.

If we discuss about the association of MGP with kidney damage assessed by common parameters of glomerular dysfunction, controversial results have been reported considering whether the levels of dp-ucMGP and ucMGP were increased or decreased. In patients with coronary artery disease, lower circulating ucMGP levels were correlated with a decrease in eGFR, but albumin-to-creatinine ratio remained unaffected [41]. In contrast, dp-ucMGP levels were progressively increased with decreasing renal function assessed by eGFR [18]. This outcome was in congruence with the findings of Schurgers et al. [17] according to which glomerular dysfunction was the only independent determinant for higher plasma levels of dp-ucMGP. In addition, a positive association was found between circulating dp-ucMGP and proteinuria in patients with advanced CKD [15]. Kurnatowska et al. [15] supported the following two conflicting deductions: either kidney damage is a key determinant of vitamin K deficiency in vasculature, or poor vitamin K status could be a risk factor for kidney dysfunction. Logically, a decline of vitamin K status comes first, resulting in an increased general predisposition for calcification, lithiasis and consequently impaired kidney function. To validate these theories further longitudinal studies are needed.

The presented data allow us to support the following conclusions: high dp-ucMGP levels could be considered a surrogate marker for VC and cardiovascular risk in CKD patients, reflecting a poor vitamin K status [17]. On the contrary, circulating ucMGP do not reflect vitamin K status and warrant future study to validate its usefulness as a marker for VC in CKD. In addition, the present literature does not lend support for Gas6 to be currently designated as marker for VC or vitamin K status in CKD, but it showed potential as proxy for impaired glomerular filtration in CKD or improving early diagnosis of DN [72].

While in the previously mentioned studies dp-ucMGP levels demonstrated an increase related to CKD progression, there are divergent views on the variations of circulating OC. Gluba-Brzózka et al. [45] found that OC levels increased in early stages of CKD and progressively decreased as the disease advanced. Contrariwise, higher circulating OC was reported in post renal transplant patients with CKD stage IV compared to CKD stage III [46]. In addition, the same pattern of serum modification was noticed for %ucOC, higher levels being found as CKD progresses [9]. Even if Holden et al. [9] reported consistent associations of %ucOC with CKD stage and urinary loss of proteins, the authors emphasized that %ucOC is not a perfect marker for vitamin K status in more advanced CKD due to the underlying secondary hyperparathyroidism, which leads to increased bone turnover, all this in the context of a diminished glomerular filtration with consequent retention of OC fragments. The OC fragments are cleared mostly by the kidneys, so there is significant heterogeneity of circulating OC in patients with renal impairment, thus limiting the usefulness of OC as a marker of bone turnover.

Further study is also required to clarify why both serum ucOC and cOC levels were increased in HD patients [10]. The authors speculate that application of OC assessment in HD patients seems to be difficult and OC carboxylation might not be a useful marker for vitamin K status in patients with HD.

## 6. Conclusions

There is still a broad research area to cover with aspects related to the roles and molecular interactions of VKDPs. Although the relationship between the modifications of circulating VKDPs and the etiologies of CKD has not been sufficiently addressed, mounting evidence shows that VKDPs are useful tools to improve early diagnosis, monitor progression or identify complications of CKD. Future studies on the assessment of VKDPs in terms of blood/urine ratio, as well as the use of mediation analyses to study the influence of eGRF in the relationship between VKDPs and CKD etiology are needed to emphasize the added benefit of VKDPs. In this respect, dp-ucMGP is more likely to become a suitable marker for detection of VC and cardiovascular risk in CKD patients.

Even if substantial efforts are still entailed to warrant the accurate validation and reproducibility for clinical implementation, researchers should stay abreast of the recent literature as VKDPs could become a common screening tool for different CKD settings in the near future.

## Figures and Tables

**Table 1:** Summary of searching strategy.

Protein	Search Term	Date	Number of Results		Number of Studies Included
			By Search Term	Total	
MGP	“Matrix Gla protein AND kidney disease”	15 August 2018	132	224	31
	“MGP AND kidney disease”	2 September 2018	92		
OC	“Osteocalcin AND kidney disease”	19 September 2018	235	470	29
	“Osteocalcine AND kidney disease”	19 September 2018	235		
Gas6	“Gas6 AND kidney disease”	2 September 2018	34	63	16
	“Growth arrest specific protein 6 AND kidney disease”	2 September 2018	29		
GRP	“GRP AND kidney disease”	2 September 2018	38	45	1
	“Gla rich protein AND kidney disease”	2 September 2018	5		
	“Gla-rich protein AND kidney disease”	2 September 2018	2		

Note: Search terms used for each protein, dates at which the databases were accessed and the results retrieved, number of results retrieved, number of studies included in review according to inclusion and exclusion criteria. Abbreviations: MGP, matrix Gla protein; OC, osteocalcin; Gas6, growth arrest specific protein 6; GRP, Gla-rich protein.

**Table 2:** Human studies on circulating MGP.

Reference	Study Type	Number of Patients and Disease State	MGP Conformations	MGP Variation vs. Controls
Schlieper et al. 2011 [12]	cross-sectional	188, HD	dp-ucMGP dp-cMGP	Both higher
Meuwese et al. 2015 [16]	cross-sectional	97, HD	t-ucMGP	Lower
Schurgers et al. 2010 [17]	prospective cohort	107, CKD stages II–V and HD	dp-ucMGP	Higher
Puzantian et al. 2018 [18]	prospective cohort	137, CKD stages II–V	dp-ucMGP	Higher
Fain et al. 2018 [19]	cross-sectional	37, HD	dp-ucMGP	Higher
Westenfeld et al. 2012 [20]	interventional	53, HD	dp-ucMGP	Higher
Mansour et al. 2017 [21]	interventional	60, Renal transplant	dp-ucMGP	Higher
Jansz et al. 2018 [22]	cross-sectional	82, HD; 31, peritoneal dialysis; 36, Renal transplant	dp-ucMGP	Lower than HD
Boxma et al. 2012 [23]	prospective cohort	60, Renal transplant	dp-ucMGP	Higher
Keyzer et al. 2015 [24]	prospective cohort	518, Renal transplant	dp-ucMGP	Higher
Cranenburg et al. 2009 [25]	cross-sectional	40, HD	ucMGP	Lower
Shroff et al. 2008 [26]	cross-sectional	61, HD	ucMGP	Lower

Abbreviations: MGP, matrix Gla protein; dp-ucMGP, dephospho-uncarboxylated MGP; dp-cMGP, dephospho-carboxylated MGP; t-ucMGP, total-uncarboxylated MGP; ucMGP, uncarboxylated MGP; HD, hemodialysis; CKD, chronic kidney disease; vs, versus.

**Table 3:** In vitro studies concerning MGP.

Reference	Type of Cells	Findings
Willy et al. 2018 [29]	Supernatants from calcifying VSMCs incubated in serum of HD patients	Lower in HRO group than HF group
Willy et al. 2017 [30]	Supernatants from calcifying VSMCs incubated in serum of HD patients	Lower in MCO group than HF group Lower in HCO group than HF group
Khan et al. 2014 [31]	Induced nephrolithiasis on MDCK cells culture	Increased MGP expression
Lu et al. 2013 [32]	Kidneys of hyperoxaluric rats	Increased MGP expression

Abbreviations: MGP, Matrix Gla protein; HRO, High Retention Onset dialysis; HF, conventional High Flow dialysis; HCO, High Cut-Off dialysis; MCO, Medium Cut-Off dialysis; MDCK, Madin-Darby Canine Kidney.

**Table 4:** Assessment of MGP in tissues.

Reference	Pathology	MGP Conformations	Findings
Lomashvili et al. 2011 [33]	Induced renal failure with VC (rats)	cMGP, ucMGP	Both had increased expression in calcified aortic VSMCs
Lorenzen et al. 2012 [34]	Renal allograft calcification (humans)	MGP	Increased expression versus non-calcified allografts
Kramann et al. 2013 [35]	Calcific uremic arteriolopathy (humans)	ucMGP	Increased expression in skin
Shroff et al. 2008 [36]	HD (humans)	cMGP, ucMGP	Increased expression in calcified blood vessels
Wei et al. 2016 [37]	Renal tissue from CKD patients vs. healthy donors (humans)	cMGP, ucMGP	Both were present in calcified renal tissue

Abbreviations: MGP, matrix Gla protein; VC, vascular calcification; ucMGP, uncarboxylated MGP; dp-ucMGP, dephospho-uncarboxylated MGP; HD, hemodialysis; vs, versus.

**Table 5:** Variations of serum OC levels in human studies.

Reference	Type of Study	Number of Patients and Disease State	OC Conformation	Findings
Holden et al. 2010 [9]	cross-sectional	172, CKD stages III–V	%ucOC	Higher as CKD progresses, associated with CKD stage
Gluba- Brzózka et al. 2016 [45]	cross-sectional	80, CKD stages I–V	Intact OC	Non-significant decreasing trend as CKD advance
Kovesdy et al. 2011 [46]	prospective cohort	639, Post renal transplant with CKD stages III–IV	Intact OC	Higher in post renal transplant patients with CKD stage IV than CKD stage III

Abbreviations: OC, osteocalcin; %ucOC, percentage of total OC that is uncarboxylated; CKD, chronic kidney disease.

**Table 6:** Interventional studies on OC.

Reference	Number of Subjects and Pathology	Drug/Treatment	Findings
Krause et al. 2018 [51]	22 patients, HD	Partial body cutaneous exposure to UVB radiation	Reduced in serum
Ma et al. 2017 [52]	31 patients with HD	Partial parathyroidectomy	Reduced in serum
Kettler et al. 2018 [59]	1059 patients with hyperphosphatemic CKD	Sucroferric oxyhydroxide, Sevelamer carbonate (phosphate binders)	Increased in serum
Mirfatahi et al. 2018 [60]	34 patients with HD	Flaxseed oil (omega-3 fatty acid and alpha-linolenic acid)	No significant change in serum
Greeviroj et al. 2018 [61]	10,031 patients with HD (meta-analysis)	Cinacalcet (calcimimetic)	No significant change in serum
Schwarz et al. 2011 [62]	58 patients with hyperparathyroidism after renal transplant	Cinacalcet	No significant change in serum
Hirai et al. 2010 [63]	47 patients with HD	Cinacalcet	No significant change in serum
Shigematsu et al. 2010 [64]	145 patients with HD	Lanthanum carbonate (phosphate binder)	No significant change in serum
Malluche et al. 2008 [65]	65 patients with HD	Lanthanum carbonate	No significant change in serum
Gomes et al. 2017 [66]	39 patients with non-dialysis dependent CKD	Aerobic exercise	No significant change in serum for cOC and ucOC
Watanabe et al. 2017 [67]	Osteoclast cell culture in mice	Indoxyl sulfate (uremic toxin)	Suppress expression
Gauthier-Bastien et al. 2014 [68]	Induced CKD by subtotal nephrectomy in mice	Calcium and phosphate diet, with vitamin D supplementation	De novo expression in VSMCs
Troib et al. 2016 [69]	Induced CKD by subtotal nephrectomy in rats	Endurance exercise	Improved expression in epiphyseal growth plate

Abbreviations: OC, osteocalcin; HD, hemodialysis; UVB, ultraviolet B; CKD, chronic kidney disease; cOC, carboxylated OC; ucOC, uncarboxylated OC; VSMCs, vascular smooth muscle cells.

## Abbreviations

%ucOC	Percentage of total osteocalcin that is uncarboxylated
CCRC	Clear cell renal carcinoma
CKD	Chronic kidney disease
cOC	Carboxylated osteocalcin
cMGP	Carboxylated matrix Gla protein
DN	Diabetic nephropathy
dp-cMGP	Dephospho-carboxylated matrix Gla protein
dp-ucMGP	Dephospho-uncarboxylated matrix Gla protein
ESRD	End-stage renal disease
Gas6	Growth-arrest specific protein 6
Gla	Carboxy glutamic acid
Glu	Glutamic acid
GRP	Gla-rich protein
HCO	High Cut-Off dialysis
HD	Hemodialysis
HF	Conventional High Flow dialysis
HRO	High Retention Onset dialysis
MCO	Medium Cut-Off dialysis
MDCK	Madin-Darby Canine Kidney
MGP	Matrix Gla protein
OC	Osteocalcin
p-cMGP	Phospho-carboxylated matrix Gla protein
Ser	Serine
t-ucMGP	Total-uncarboxylated matrix Gla protein
ucMGP	Uncarboxylated matrix Gla protein
ucOC	Uncarboxylated osteocalcin
VC	Vascular calcification
VKDPs	Vitamin K dependent proteins
VSMCs	Vascular smooth muscle cells
